# Attitudes towards organ donation in Syria: a cross-sectional study

**DOI:** 10.1186/s12910-020-00565-4

**Published:** 2020-12-09

**Authors:** Mario Tarzi, Malke Asaad, Joudi Tarabishi, Obada Zayegh, Rama Hamza, Ahmad Alhamid, Aya Zazo, Mohamad Morjan

**Affiliations:** Faculty of Medicine, Aleppo University, Al-Mouhafaza, Aleppo, Syria

**Keywords:** Organ, Donation, Transplant, Deceased, Brain death, Syria

## Abstract

**Background:**

The perception of organ donation and brain death among Syrian population has not been previously explored. The goal of this study is to evaluate the attitude and knowledge of organ donation among Syrians and the willingness of this population to donate their organs.

**Methods:**

We conducted a survey-based cross-sectional study in four hospitals in Aleppo, Syria in November 2019. Patient demographic, awareness of brain death; and attitude toward organ donation were collected and analyzed.

**Results:**

A total of 350 individuals were invited to participate in the survey among whom 303 (197 females, 106 males) agreed to participate in the study (87% response rate). The majority of our participants (n = 249, 82%) heard about organ donation with television (n = 166, 55%), social media (n = 77, 25%), and the internet (n = 77, 25%) being the most common sources of information. When assessing knowledge about brain death, only 40% (n = 116) answered 3 or more questions (out of 5) correctly. Fifty-eight percent (n = 176) of respondents agreed with the idea of organ donation and 183 (62%) would like to donate their organs one day. The leading motivation to organ donation was the desire to help (n = 234, 77%), while the most common reason to refuse donation was the refusal to disfigure a dead body by removing an organ (n = 125, 41%). Religious reasons were cited as motivation for organ donation by 43% of participants (n = 130), and a reason for refusing to donate organs by 24% (n = 71). Most respondents (n = 261, 88%) were unaware of the laws and legislations related to organ donation in Syria. When asked if religion and law were encouraging organ donation, 76% of respondents (n = 226) would donate their organs. Although more positive attitude was found in those with better brain death knowledge (score ≥ 3), this did not translate into more willingness to donate organs in this group of participants.

**Conclusions:**

The promotion of organ donations from deceased donors is a necessity given the rising shortage of organs. The information provided by this study could help policy makers build future strategies to promote deceased organ donation programs and overcome current obstacles preventing such initiatives from achieving their goals.

## Background

Our organs are subject to diverse types of changes, some of which could be reversed by the organs’ regulatory systems, while others are irreversible and can lead to organ failure. Prior to the era of organ transplantation, management of several end-stage diseases was conservative without a viable alternative to the failed organ. However, remarkable progress in the fields of immunology and surgery has allowed medicine to triumph over many end-stage diseases, such as liver and kidney failure [1]. Not surprisingly, the great success of organ transplantation has yielded a significant increase of 70% in demand during the past decade [2–4]. Worldwide, the current imbalance between supply and demand in various organs (mainly liver and heart) results in over 20% of people on waiting lists dying every year before receiving the needed organ [5].

The history of organ transplantation in Syria is traced back to 1979, when the first kidney transplant was performed [6]. Later on, three cases of heart transplants were performed at Tishreen Military Hospital in the early 1990s. Two liver transplants were performed in 2017 and 2019 et al.-Assad University Hospital in Damascus. At the present time, kidney, cornea and bone marrow are the only organs being transplanted in Syria [7].

Organ transplantation in Syria mainly relied on living donors in 1980s. Since the enactment of Law number 30 in November 2003, a proper definition of “brain death” was made, and the permission of using organs from deceased donors in addition to living donors “both related and unrelated” was granted [7]. A recent update on the Law was made in 2009 which delineated the establishment of Syrian National Center for Organ transplantation [8]. However, this center has remained inactive and so has the national deceased organ donation program [6, 8].

The number of transplant surgeries performed in Syria has been significantly impacted by the ongoing war and such surgeries are currently performed in the capital, Damascus only. The number of total of kidney transplants went down from 385 (17 per million people, PMP) in 2010 to 154 in 2013 (6 PMP) [8]. There has been an increase in the rates of kidney transplants since then to reach 251 cases in 2018 (14 PMP), but it still lower than the numbers before the war (in 2010) [6]. The majority of these organs have been donated from living donors, mostly relatives, while deceased organ donation is still an unmet need. The promotion of organ donations from deceased donors is a necessity given the rising shortage of organs, but is still hinders by several obstacles. To the best of our knowledge, no study has evaluated the perception of organ donation and brain death among Syrian population. In this study, we aimed is to evaluate the attitude and knowledge of organ donation and brain death among Syrians and the willingness of this population to donate their organs. A secondary objective would be to assess the factors both precipitating and limiting organ donation. The information provided by this study could help policy makers build future strategies to promote deceased organ donation programs and overcome current obstacles preventing such initiatives from achieving their goals.

## Methods

We conducted a survey-based cross-sectional study to assess attitudes towards organ donation among Syrian population. The questionnaire was designed based on literature review and experts’ feedback [9–14]. The study was piloted on 13 individuals with no subsequent changes to the survey deemed necessary. We collected data on the following information: (1) demographic and socioeconomic characteristics of the participants; (2) knowledge and awareness of organ donation and brain death; and (3) attitude toward organ donation.

The questionnaire was delivered by trained medical students and conducted in-person with each participant. The survey was administered in November 2019, in Aleppo city, Syria. Four data collection centers were included: Aleppo University Hospital, Al-Shahba Private Hospital, Al-kalema Private Hospital, and Saint-Louis Private Hospital. We targeted all patients and visitors (inpatient and outpatient) of the study centers who were 18 years or older. We chose a combination of public and private hospitals to cover different socioeconomic classes of the Syrian society. The survey distributors explained the study to all participants. We obtained an informed verbal consent from all participants prior to participation in the study and an ethical approval from the research committee of the University of Aleppo prior to the conduction of the study. We administered the survey in Arabic which is the native language in Syria. An English and Arabic versions of the survey are presented in Additional files 1, 2.

### Statistical analysis

Categorical variables were presented as frequencies and percentages, whereas numerical variables were presented as mean ∓ standard deviation (SD). We used Chi-square test and Fisher’s exact test (when approporiate) to examine the relationship between categorical variables, and independent sample t-test to investigate the difference in mean age between groups. Five questions were asked to evaluate the knowledge of brain death. Participants were divided into two groups: brain death score ≥ 3 if they answered 3 or more questions correctly; and brain death knowledge score ≤ 2 if they answered 2 or less correct questions. A *p* value < 0.05 was considered statistically significant. Statistical analysis was performed using JMP Pro 14 software (JMP, Pro 14, SAS Institute Inc, Cary, NC, 1989-2019).

## Results

### Participants demographics

A total of 350 individuals were invited to participate in the survey among whom 303 agreed to participate in the study (87% response rate). Sixty-five percent of respondents were females (n = 197) and thirty-five percent were males (n = 106), with a mean age of 42 ∓ 15 years. The majority of the study participants were married (n = 217, 72%), without work (including retired and unemployed) (n = 160, 59%), and lived in urban (n = 229, 76%) as opposed to rural settings (n = 72, 24%). Details on the demographic and socioeconomic characteristics of the participants are shown in Table 1.

**Table 1:** Respondents demographic characteristics

	N (%)
Number of respondents	303
Age, years (mean ∓ SD)	42 ∓ 15
Gender	
Male	106 (35)
Female	197 (65)
Residency	
Urban	229 (76)
Rural	72 (24)
Marital status	
Single	59 (20)
Married	217 (72)
Divorced	7 (2)
Widow	18 (6)
Level of education	
Illiterate	32 (11)
Less than high school	144 (48)
High school	45 (15)
College	23 (8)
University	54 (18)
Current job	
Private work	34 (12)
Employed	79 (29)
Does not work	160 (59)
Chronic disease	
Yes (treated)	81 (28)
Yes (not treated)	6 (2)
No	202 (70)

### Knowledge of organ donation and brain death

The majority of our participants (n = 249, 82%) heard about organ donation with television (n = 166, 55%), social media (n = 77, 25%), and the internet (n = 77, 25%) being the most common sources of information. Health-care workers as a source of information for organ donation was reported by only 11% of respondents. When assessing knowledge about brain death, only 40% (n = 116) answered 3 or more questions (out of 5) correctly. Table 2 summarizes participants’ response to the questions related to the knowledge of organ donation and brain death.

**Table 2:** Awareness and knowledge of organ donation and brain death

	N (%)
Have you ever heard of organ donation?	
Yes	249 (82)
No	54 (18)
Where did you hear about organ donation?^a^	
Social media	77 (25)
Television	166 (55)
Newspapers and magazines	26 (9)
Internet	77(25)
Educational center	17 (6)
Friends	57 (19)
Family	40 (13)
Health-care workers	34 (11)
As far as you are concerned, what do you relate organ donation to?^a^	
Donation after death	187 (62)
Donation during life	160 (53)
Brain death	51 (19)
Organ trafficking	52 (17)
Have you ever heard of brain death?	
Yes	188 (63)
No	111 (37)
Do brain-dead patients respond if someone touched their eyes (by frowning, eye blinking, limb movement, etc.)?	
Yes	24 (8)
No	89 (30)
Do not know	186 (62)
How do brain-dead patients keep their respiratory function?	
By ventilator	137 (46)
Without the aid of any equipment	28 (9)
Do not know	134 (45)
Do brain-dead patients feel pain?	
Yes	30 (10)
No	117 (39)
Do not know	152 (51)
Is the recovery of brain-dead patients possible?	
Yes	63 (21)
No	87 (29)
Do not know	149 (50)
As far as you are concerned, what is brain death related to?^a^	
Coma	147 (49)
Vegetative state	24 (8)
Clinical death	157 (52)
Organ donation	45 (15)

^a^More than one answer was possible

### Attitude toward organ donation

When assessing participants’ attitude towards organ donation, 176 (58%) of respondents agreed with the idea of organ donation, 183 (62%) would like to donate their organs one day, and 200 (67%) would encourage organ donation. However, when asked if they would agree to donate organs of a family member after death, the percentage of those who agreed went down to 50% (n = 149), which further went down when asked whether they would donate family members organs after brain death (n = 102, 35%). Almost half of the respondents would donate organs only after death (n = 121, 51%), while 28% (n = 68) would donate anytime and 21% (n = 50) would donate only during life.

The leading motivation to organ donation was the desire to help (n = 234, 77%), while the most common reason to refuse donation was the refusal to act with corpses (n = 125, 41%). Religious reasons were cited as motivation for organ donation by 43% of participants (n = 130), and a reason for refusing to donate organs by 24% (n = 71). Most respondents (n = 261, 88%) were unaware of the laws and legislations related to organ donation in Syria. If religion and law were encouraging organ donation, 76% of respondents (n = 226) would donate their organs. Respondents’ attitudes toward organ donation are summarized in Table 3.

**Table 3:** Attitude towards organ donation

	N (%)
What is your attitude toward organ donation?	
Agree	176 (58)
Disagree	76 (25)
Do not know	50 (17)
Would you like to donate an organ or more one day?	
Yes	183 (62)
No	112 (38)
When would you prefer to donate your organs?	
Only during life	50 (21)
Only after death	121 (51)
Any time	68 (28)
Would you agree to donate organs of a family member after their death?	
Yes	149 (50)
No	148 (50)
Would you agree to donate organs of a family member in cases of brain death?	
Yes	102 (35)
No	191 (65)
Would you encourage organ donation?	
Yes	200 (67)
No	99 (33)
Who would you want to donate your organs to?	
Only for relatives	94 (35)
Only for non-relatives	1 (0.3)
Relatives and non-relatives	174 (65)
In case you agreed to donate your organs, what would your motivations be?	
Financial	5 (2)
Religious	130 (43)
It does not harm, so why not?	125 (41)
The desire to help	234 (77)
In case you disagreed to donate your organs, what would the reasons be?	
Absence of financial benefit	5 (2)
Religious beliefs	71 (24)
Social and familial barriers	88 (29)
Refusing to act with corpses	125 (41)
Fear of being murdered in order to obtain organs	117 (39)
Fear of not receiving a good medical care	104 (34)
Fear of talking about death	29 (10)
Lack of knowledge about organ donation	75 (25)
Organs recipients are not chosen fairly	121 (40)
Are you aware of the laws and legislations related to organ donation, brain death and organ transplantation in Syria?	
Yes	37 (12)
No	261 (88)
If religion and law were encouraging organ donation, would you do it?	
Yes	226 (76)
No	70 (24)
Do you have an experience with organ donation?	
Yes	2 (1)
No	297 (99)
If we conducted a lecture about organ donation, would you attend it?	
Yes	87 (29)
No	215 (71)

### Relationship between brain death knowledge and organ donation

Participants demographics were similar between those who scored brain death score ≥ 3 and brain death knowledge score ≤ 2 except higher education level in those who scored ≥ 3. Although more positive attitude was found in those with better brain death knowledge (score ≥ 3), this did not translate into more willingness to donate organs in this group of participants. Demographics and attitude toward organ donation by brain death knowledge are summarized in Table 4.

**Table 4:** Attitude towards organ donation by brain death knowledge

	Brain death knowledge score ≤2 N (%)	Brain death knowledge score 3≥ N (%)	Total N (%)	*p* value
No. (%)	176(60)	116(40)	292	
Age, years (mean ∓ SD)	42 ∓ 15	41 ∓ 15		0.55
Gender				
Male	58 (33)	44 (38)	102 (35)	0.38
Female	118 (67)	72 (62)	190 (65)	
Residency				
Urban	130 (74)	90 (78)	220 (76)	0.44
Rural	45 (26)	25 (22)	70 (24)	
Education				
Illiterate/less than high school	119 (69)	51 (44)	70 (59)	< .0001*
High school or higher	53 (31)	64 (66)	117 (41)	
Current job				
Does not work	99 (62)	55 (53)	154 (59)	0.13
Works	60 (83)	49 (47)	109 (41)	
Marital status				
Not married	44 (25)	37 (32)	81 (28)	0.18
Married	132 (75)	78 (68)	210 (72)	
Chronic diseases				
Not treated	113 (69)	81 (70)	194 (70)	0.84
Absent or treated	50 (31)	34 (30)	84 (30)	
What is your attitude toward organ donation?				
Agree	96 (55)	74 (64)	170 (58)	0.02*
Disagree	43 (24)	32 (28)	75 (26)	
Do not know	37 (21)	10 (9)	47 (16)	
Would you like to donate an organ or more one day?				
Yes	113 (65)	66 (57)	179 (62)	0.17
No	60 (35)	49 (43)	109 (38)	
When would you prefer to donate your organs?				
Only during life	31 (22)	18 (21)	49 (21)	0.78
Only after death	70 (49)	46 (53)	116 (51)	
Any time	42 (29)	22 (26)	64 (28)	
Would you agree to donate organs of a family member after their death?				
Yes	86 (50)	59 (51)	145 (50)	0.89
No	86 (50)	57 (49)	143 (50)	
Would you agree to donate organs of a family member in cases of brain death?				
Yes	55 (32)	42 (37)	97 (34)	0.43
No	115 (68)	72 (63)	187 (66)	
Would you encourage organ donation?				
Yes	111 (64)	81 (70)	192 (66)	0.32
No	62 (36)	35 (30)	97 (34)	
Are you aware of the laws and legislations related to organ donation, brain death and organ transplantation in Syria?				
Yes	18 (10)	17 (15)	35 (12)	0.25
No	156 (90)	97 (85)	253 (88)	
If religion and law were encouraging organ donation, would you do it?				
Yes	127 (73)	91 (80)	218 (76)	0.21
No	46 (27)	23(20)	69(24)	

^*^Statistically significant

### Relationship between desire to donate and participants’ demographics

Markedly, neither socioeconomic and demographic factors nor knowledge of brain death were significantly associated with willingness to donate. However, donation patterns differed between those who are willing to donate and those not. Table 5 represents the factors related to willingness to donate organs.

**Table 5:** Attitude towards organ donation by desire to donate organs

	Desire N (%)	No desire N (%)	Total N (%)	*p* value
No. (%)	183 (62)	112 (38)	295	
Age, years (mean ∓)	43 ± 15	40 ± 14		0.13
Gender				
Male	63 (34)	39 (35)	102 (35)	0.94
Female	120 (66)	73 (65)	193 (65)	
Residency				
Urban	143 (78)	81 (74)	224 (76)	0.38
Rural	40 (22)	29 (26)	69 (24)	
Education				
Illiterate or less than high school	112 (62)	61 (55)	173 (59)	0.24
High school or higher	69 (38)	50 (45)	119 (41)	
Current job				
Does not work	99 (61)	57 (55)	156 (59)	0.35
Works	63 (39)	46 (45)	109 (41)	
Marital status				
Not married	53 (29)	30 (27)	83 (28)	0.72
Married	130 (71)	81 (73)	211 (72)	
Chronic diseases				
Not treated	114 (66)	83 (75)	197 (70)	0.10
Absent or treated	58 (34)	27 (25)	85 (30)	
Have you ever heard of organ donation?				
Yes	152 (83)	90 (80)	242 (82)	0.56
No	31 (17)	22 (20)	53 (18)	
Brain death knowledge score				
Brain death knowledge score ≤ 2	113 (63)	60 (55)	173 (60)	0.17
Brain death knowledge score ≥ 3	66 (37)	49 (45)	115 (40)	
When would you prefer to donate your organs?				
Only during life	41 (23)	8 (15)	49 (21)	< .0001*
Only after death	76 (42)	39 (75)	115 (50)	
Any time	63 (35)	5 (10)	68 (29)	
Would you agree to donate organs of a family member after their death?				
Yes	120 (66)	28 (25)	148 (51)	< .0001*
No	61 (34)	83 (75)	144 (49)	
Would you agree to donate organs of a family member in cases of brain death?				
Yes	87 (48)	14 (13)	101 (35)	< .0001*
No	94 (52)	93 (87)	187 (65)	
Would you encourage organ donation?				
Yes	157 (86)	38 (35)	195 (67)	< .0001*
No	26 (14)	71 (65)	97 (33)	
Who would you want to donate your organs to?				
Only for relatives	48 (26)	46 (56)	94 (36)	< .0001*
Only for strangers	1 (1)	0 (0)	1 (0)	
To anyone	132 (73)	36 (44)	168 (64)	
Are you aware of the laws and legislations related to organ donation, brain death and organ transplantation in Syria?				
Yes	23 (13)	14 (13)	37 (13)	1.00
No	158 (87)	96 (87)	254 (87)	
If religion and law were encouraging organ donation, would you do it?				
Yes	167 (91)	54 (50)	221 (76)	< .0001*
No	16 (9)	53 (50)	69 (24)	

^*^Statistically significant

## Discussion

Exploring the attitude of people towards organ donation is of vital importance in developing strategies to increase social awareness of organ donation and eliminate any fears associated with this process. In our study, we found that the majority of our participants are willing to donate their organs, driven by the pure desire to help others, both relatives and non-relatives. We did not identify a significant association between brain death’s knowledge and the willingness to donate organs. Most respondents were unaware of the laws and legislations related to organ donation in Syria and if the latter two were encouraging organ donation, more respondents are likely to donate their organs. Addressing misconceptions through awareness programs could potentially close the current gap between donors and recipients.

The results of our survey reveal that the majority of the participants (82%) heard about organ donation. This corresponds with the results of prior studies [9, 12, 15]. A study from Egypt found that 89% of the participants have heard of organ donation, while 53% of them could recognize the organs that could be transplanted [9]. Our findings reflect that the majority (62%) of the study group was willing to donate their organs. This percentage is higher than that in Qatar (35.7%), Egypt (44%), and Nigeria (29.7%) [2, 9, 12], similar to that of Jordan (67%) and Pakistan (62%) [16, 17] and lower than its counterpart in the US (98%) [18]. Although the majority tends to donate their organs to anyone in need on altruistic motive, almost 35% would donate only to their relatives. “Relatives are priority when doing favor” is what many of the participants have referred to when explaining their point. Preference to donate organs to relatives has also been reported in other countries like Egypt [9], Brazil [19] and Saudi Arabia [10]. Relatives’ impact would exceed the former point to a new one, which is the tendency to donate the organs of their beloved ones after death but not after being diagnosed with brain death. It can be explained with the misunderstanding of the concept of brain death, as 21% of the study group believe that recovery could happen for a brain-dead patient [20–22]. The previous result highlights the role of health facilities in explaining the correct definition of brain death and how donated organs can be the glimpse of hope for other people in need.

Several sources can serve to educate individuals about organ donation. In our study, television seems to be the main method of knowledge about organ donation. The television, social media, and the internet have permitted sharing of medical information and provided many platforms for the purpose of promoting the general public health. This could be interpreted by the relatively low cost, ease of usage and reach for many people compared to other sources of information. This seems to be the case in other countries as well [9, 10]. However, the credibility of the information provided on these platforms are questionable and need further validation. On the other hand, Health Care Workers (HCWs) were the source of information in only 11% of the respondents despite being a more accurate source of information [9], and their interactions could influence the final decision of the family [23, 24]. HCW should be targeted by government policies to be the major source of information when it relates to organ donation. This can be applied by engaging the HCWs’ system in specific workshops on various topics of interest and emphasizing the value of providing them with up-to-date information. Setting websites managed by HCWs for the public to refer to whenever they have questions could be a useful step as well.

We evaluated various factors affecting the willingness of organ donation. The pure desire to help followed by religious reasons were the two most frequent reasons for donating organs while financial reasons were only motivating for 2% of respondents. This could be explained by the belief that getting paid for good actions curtail the good deed and that profiting from organ donation is another form of organ trafficking.

A study from Jordan has found that religious (36%) and lack of financial incentive (44%) constitute important reasons to refuse organ donation [13]. Another study from Saudi Arabia reflected that humanity (68%) and religious (62%) reasons are the most important to accept organ donation, while money has only accounted for 0.6% [10]. In the same study, religion was a reason for refusing to donate organs in 27.5% of respondents.

Maintenance of body’s integrity is one of the main concerns of our study group, reflected by “Refusing to act with corpses” (disfiguring the body of the donor following death in order to obtain organs) being chosen as the most common cause for refusal to donate organs. This idea goes in line with what Sanner et al. has stated, “those who are in favor of cremation and those who are not concerned about the disfiguration of the body tend to have a favorable attitude toward organ donation” [25]. People should be informed that the process of organ donation is handled in an operative setting by professional doctors who take the best care of the deceased patient and following which the body is returned to the family for a possible Open Casket funeral. Mistrust in medical staff, represented by fear of being murdered to obtain organs and bias in choosing recipients, constituted other major reasons for the disapproval of organ donation. Some also has the misconception that removing an organ might result in the donor’s death. The psychologic effects presented by fear and insecurity, that prevail at times of war could potentially exacerbate patients’ mistrust in the medical system. Governmental interventions should focus on addressing patient mistrust and misinformation about organ donation in the hope of increasing the number of those willing to donate. Clarity and transparency in organ distribution among all citizens of different financial and social status are needed to ensure equal opportunities in receiving organ transplantations. Seventeen percent of the respondents related organ donation to organ trafficking. This fear could have been fueled by several reports of organ traffickers preying on Syrian refugees [26, 27]. However, no studies or official numbers exist in that regard.

Understanding factors related to organ donation is key in any policy intervention trying to address this issue. Brain death knowledge is a fundamental factor that is correlated deeply with organ donation, as the majority of organs donated are those of brain-dead patients [28]. Our study depicts that 40% of our participants had good knowledge of brain death, yet almost half of the participants relate brain death to coma, which illustrates a profound misunderstanding of the pathophysiology of brain death. Participants with better brain death knowledge demonstrated a favorable attitude towards organ donation, but they were not more likely to donate their organs. This is in contrast to many studies that showed correspondence between the two entities [29, 30]. Mistrust in the practical application of organ donation could potentially explain the lack of association in our study.

Future projects aiming to increase the rates of organ donation in Syria should take into consideration the importance of “family pattern” when it comes to sign the final decision of organ donation, the necessity of scientific sessions about drain death and organ donation to dispel community myths and encourage people to donate. The role of transparency in choosing recipients is vital in ways of increasing the trust in the noble role of the medical team. Individuals could be asked routinely about their willingness to donate during application to official documents from the government (e.g. driver license) with available educational resources for those who need more information prior to making a decision [9]. Religious and legal factors play a critical role in the decision for organ donation as more people might be willing to donate if religion and law were encouraging organ donation. Interestingly, both law and religion in Syria allow for organ donation. The enactment of law number “30” defines an important landmark in the progress of organ donation legalization process in Syria. It characterized the concept of brain death, allowed the donation of deceased organs and expanded living organ donation to both related and unrelated recipients [7]. Moreover, Islam and Christianity, the two main religions in Syria, stand for organ donation and permit it for the benefit of people in need. Therefore, churches and mosques could play an important role in informing people about the position of both Islam and Christianity from organ donation. In addition, awareness programs are crucial to educate people since only 12% are aware of laws related to organ donation in Syria. Distinguishing organ donation during from that after life and addressing the misconceptions associated with deceased organ donation could help improve the rates of those willing to donate after life. The current lack of a national organ donation registry limits the ability to match donors with those in need [6]. Providing donors with a donor card or having an app that match donors with recipients would streamline the process of organ donation and improve access for patients in need.

There are several limitations in our study that worth highlighting. The survey we used was not validated given the lack of validated survey for assessment of organ donation. This may have introduced bias in our study. Our study assessed 4 hospitals in one Syrian city, and the results might not be generalizable to other settings. However, we included hospitals from various areas of the city belonging to both the private and public sectors to ensure the diversity and representability of our sample. Finally, patients and their companions could be more likely to favor organ donation compared to the general population, given their health or their relatives health status. Future studies should assess the efficacy of various interventions to improve awareness of organ donations and willingness to donate organs, during and after life.

## Conclusions

The promotion of organ donations from deceased donors is a necessity given the rising shortage of organs. We found that the majority of our participants are willing to donate their organs, driven by the pure desire to help others. Fear of disfiguring a dead body by removing an organ and mistrust in the recipients’ selection process were the main deterrents of organ donation. We did not find significant association between brain death’s knowledge and the willingness to donate organs. The information provided by this study could help policy makers build future strategies to promote deceased organ donation programs and overcome current obstacles preventing such initiatives from achieving their goals.

## Supplementary Information

**Additional file 1**. English version of the survey.

**Additional file 2**. Arabic version of the survey.

## Data Availability

The data that support the findings of this study are available on request by the journal. The data are not publicly available due to institution research privacy policy.

## Abbreviations

PMP: Per million people
HCWs: Health care workers
