# Supplementary material for: Attitudes towards organ donation in Syria: a cross-sectional study
Source: BMC Med Ethics. 2020 Dec 9;21:123. doi: 10.1186/s12910-020-00565-4 (PMC7727146; doi:10.1186/s12910-020-00565-4)
Supplement: Supplementary file 1 — Additional file 1. English version of the survey. [file 12910_2020_565_MOESM1_ESM.docx]

**Survey**

**Respondents demographic characteristics:**

1. Age:
2. Gender:
   - Male
   - Female
3. Residency:
   - Urban
   - Rural
4. Level of education:
   - Illiterate
   - Less than high school
   - High school
   - College
   - University
5. Current job:
   - Private work
   - Employed
   - Does not work
   - Other:………
6. Marital Status:
   - Single
   - Married
   - Divorced
   - Widow
7. Do you suffer from a chronic disease (cardiac, endocrine, renal, pulmonary, diabetes, hypertension, tumor)?
   - Yes (treated)
   - Yes (not treated)
   - No

**Awareness and knowledge of organ donation:**

1. Have you ever heard about organ donation?
   - Yes
   - No
2. Where did you hear about organ donation? (you may choose more than *one* answer)
   - Social Media (Facebook, WhatsApp, Instagram…etc.)
   - Television
   - Newspapers and magazines
   - Internet
   - Educational center
   - Friends
   - Family
   - Health-care workers (doctor, nurse, technician…etc.)
3. As far as you are concerned, what do you relate organ donation to? (you may choose more than *one* answer)
   - Donation after death
   - Donation during life
   - Brain death
   - Organ trafficking

**Awareness and knowledge of brain death:**

1. Have you ever heard of brain death?
   - Yes
   - No
2. Do brain-dead patients respond if someone touched their eyes (by frowning, eye blinking, limb movement…etc.)?
   - Yes
   - No
   - Do not know
3. How do brain-dead patients keep their respiratory function?
   - By ventilator
   - Without the aid of any equipment
   - Do not know
4. Do brain-dead patients feel pain?
   - Yes
   - No
   - Do not know
5. Is the recovery of brain-dead patients possible?
   - Yes
   - No
   - Do not know
6. As far as you are concerned, what is brain death related to? (you may choose more than *one* answer)
   - Coma
   - Vegetative state
   - Clinical death
   - Organ donation

**Attitude towards organ donation:**

1. What is your attitude towards organ donation?
   - Agree
   - Disagree
   - Do not know
2. Would you like to donate an organ or more one day?
   - Yes
   - No
3. When would you like to donate your organs?
   - Only during life
   - Only after death
   - Any time
4. Would you agree to donate organs of a family member after their death?
   - Yes
   - No
5. Would you agree to donate organs of a family member in cases of brain death?
   - Yes
   - No
6. Would you encourage organ donation?
   - Yes
   - No
7. Whom would you donate your organs to?
   - To relatives only
   - To non-relatives only
   - Relatives and non-relatives
8. If you agreed to donate your organs, what would your motivations be? (you may choose more than *one* answer)
   - Financial
   - Religious beliefs
   - It does not harm, so why not?
   - The desire to help
9. If you disagreed to donate your organs, what would the reasons be? (you may choose more than *one* answer)
   - Absence of financial benefit
   - Religious beliefs
   - Social and familial barriers
   - Refusing to act with corpses
   - Fear of being murdered in order to obtain organs
   - Fear of not receiving a good medical care
   - Fear of talking about death
   - Lack of knowledge about organ donation
   - Organ recipients are not chosen fairly
10. Are you aware of the laws and legalizations related to organ donation, brain death and organ transplantation in Syria?
    - Yes
    - No
11. If religion and law were encouraging organ donation, would you do it?
    - Yes
    - No
12. Do you have an experience with organ donation?
    - Yes
    - No
13. If we conducted a lecture about organ donation, would you attend it? Please provide us with your email or phone number in case of agreement.

…………………………………………….
