# Supplementary material for: Attitudes towards organ donation in Syria: a cross-sectional study
Source: BMC Med Ethics. 2020 Dec 9;21:123. doi: 10.1186/s12910-020-00565-4 (PMC7727146; doi:10.1186/s12910-020-00565-4)
Supplement: Supplementary file 2 — Additional file 2. Arabic version of the survey. [file 12910_2020_565_MOESM2_ESM.docx]

**ثقافة التبرع بالأعضاء و الموت الدماغي لدى مراجعي مشفى حلب الجامعي وموقفهم من التبرع**

**معلومات عامة:**

1. العمر: ....................
2. الجنس:
    🞎 ذكر 🞎 أنثى
3. السكن:
    🞎 الريف 🞎 المدينة
4. المستوى الدراسي:
    🞎 لا أقرأ ولا أكتب 🞎 أقل من الثانوي 🞎 ثانوي 🞎 معهد 🞎 كلية جامعية
5. العمل الحالي:
    🞎 صاحب\ة مشروع 🞎 موظف\ة 🞎 عاطل\ة عن العمل 🞎 أخرى:............
6. الحالة الاجتماعية:

🞎 عازب\ة 🞎 متزوج\ة 🞎 مطلق\ة 🞎 أرمل\ة

1. هل تعاني من أمراض مزمنة (قلبية، غدية، كلوية، تنفسية، سكري، ضغط، أورام)؟ 🞎 نعم، معالج 🞎 نعم، غير معالج 🞎 كلا

**التبرع بالأعضاء كمفهوم:**

1. هل سمعت يوماً عن التبرع بالأعضاء؟

🞎 نعم 🞎 كلا

1. أين سمعت عن التبرع بالأعضاء (يمكن اختيار أكثر من خيار) ؟

🞎 السوشال ميديا (فيسبوك، واتساب، انستاغرام....الخ) 🞎 التلفاز 🞎الصحف و المجلات 🞎 الانترنت 🞎 مركز تعليمي 🞎 صديق 🞎العائلة 🞎 عاملين في المجال الطبي (طبيب، ممرض، تقني....الخ)

1. بماذا يرتبط مفهوم التبرع بالأعضاء حسب وجهة نظرك (يمكن اختيار أكثر من خيار) ؟ 🞎التبرع بعد الوفاة 🞎التبرع أثناء الحياة 🞎 الموت الدماغي 🞎 تجارة الأعضاء

**الموت الدماغي كمفهوم:**

1. هل سمعت يوماً عن الموت الدماغي؟

🞎 نعم 🞎 كلا

1. هل يقوم الشخص في حالة الموت الدماغي بالرد (عبوس، طرف عين، تحريك طرف) في حال قام أحدهم بلمس العين؟
   🞎 نعم 🞎 كلا 🞎 لا ادري
2. كيف يحافظ الشخص في حالة الموت الدماغي على وظيفة التنفس؟

🞎 بواسطة المنفسة 🞎 دون مساعدة أجهزة 🞎 لا أدري

1. هل يشعر الشخص في حالة الموت الدماغي بالألم؟

🞎 نعم 🞎 كلا 🞎 لا أدري

1. هل قد يتعافى الشخص في حالة الموت الدماغي؟

🞎 نعم 🞎 كلا 🞎 لا أدري

1. بماذا يرتبط مفهوم الموت الدماغي حسب وجهة نظرك (يمكن اختيار أكثر من خيار) ؟

🞎 السبات Coma 🞎الحالة الانباتية 🞎 الموت السريري 🞎 التبرع بالأعضاء

**موقفك من التبرع بالأعضاء:**

1. ما هو موقفك من التبرع بالأعضاء؟

🞎 أوافق 🞎 لا أوافق 🞎 لا أعلم

1. هل ترغب في التبرع بعضو واحد أو أكثر في يوم ما؟

🞎 نعم 🞎 كلا

1. متى ترغب بالقيام بالتبرع؟

🞎 أثناء الحياة فقط 🞎 بعد الوفاة فقط 🞎 في أي وقت

1. هل قد توافق على التبرع بعضو أو أعضاء أحد أقاربك بعد وفاته؟

🞎 نعم 🞎 كلا

1. هل قد توافق على التبرع بعضو أو أعضاء أحد أقاربك الذي يعاني من حالة موت دماغي؟
    🞎 نعم 🞎 كلا
2. هل قد تشجع التبرع بالأعضاء؟
    🞎 نعم 🞎 كلا
3. لمن قد ترغب بالتبرع بأعضائك؟
    🞎 للأقارب فقط 🞎 للغريب فقط 🞎للقريب أو الغريب
4. في حال الموافقة على التبرع بالأعضاء، ما هو السبب الذي يدفعك للقيام بذلك (يمكن اختيار أكثر من خيار) ؟

🞎 مردود مالي 🞎 معتقدات دينية 🞎 لن تضرني، فلم لا؟ 🞎 النية الصافية لمساعدة المحتاج

1. في حال رفض التبرع بالأعضاء، ما هو سبب عدم القيام بعملية التبرع (يمكن اختيار أكثر من خيار)؟

🞎 غياب المردود مالي 🞎 معتقدات دينية 🞎 تأثير الأهل و المجتمع 🞎 رفض فكرة التمثيل بالجثة بعد الوفاة 🞎الخوف من القتل أو إحداث الوفاة بغية الحصول على الأعضاء 🞎 الخوف من عدم الحصول على العناية الطبية اللازمة 🞎الخوف من التكلم عن الموت 🞎نقص المعرفة حول التبرع بالأعضاء 🞎عدم نزاهة اختيار المرضى المستفيدين من الأعضاء

1. هل أنت مدرك للقوانين والتشريعات الخاصة بالتبرع بالأعضاء، الموت الدماغي، و زرع الأعضاء في بلدك؟
    🞎 نعم 🞎 كلا
2. في حال كان القانون والدين يشجعك على القيام بالتبرع بالأعضاء، فهل تقوم به؟
   🞎 نعم 🞎 كلا
3. هل لديك تجربة مع التبرع بالأعضاء؟
   🞎 نعم 🞎 كلا

................................................................................................................

1. في حال قمنا بإلقاء محاضرة عن " التبرع بالأعضاء"، فهل ترغب بالمشاركة؟
